# RGS14 binds to GNAI3 and regulates the proliferation and apoptosis of human spermatogonial stem cells by affecting PLPP2 expression and MAPK signaling

**DOI:** 10.3389/fcell.2025.1593595

**Published:** 2025-04-25

**Authors:** Bang Liu, Aimin Deng, Lvjun Liu, Lin peng, Xiaowen Liu, Xiangyu Chen, Fang Zhu, Shusheng Zhang, Dai Zhou

**Affiliations:** ^1^ Hunan Provincial Key Laboratory of Regional Hereditary Birth Defect Prevention and Control, Changsha Hospital for Maternal and Child Health Care Affiliated to Hunan Normal University, Changsha, Hunan, China; ^2^ School of Basic Medicine Science, Institute of Reproduction and Stem Cell Engineering, Central South University, Changsha, Hunan, China; ^3^ Reproductive and Genetic Hospital of CITIC-Xiangya, Changsha, Hunan, China; ^4^ The First Hospital of Changsha, The Affiliated Changsha Hospital of Xiangya School of Medicine, Central South University, Changsha, Hunan, China

**Keywords:** RGS14, GNAI3, PLPP2, spermatogonial stem cells, NOA, proliferation

## Abstract

**Background:**

Non-obstructive azoospermia (NOA) represents a severe form of male infertility, characterized by the absence of sperm in the ejaculate due to impaired spermatogenesis. Spermatogonial stem cells (SSCs), which ensure continuous sperm production, are critical for maintaining male fertility. Despite their importance, the molecular mechanisms governing SSC fate determination and their role in NOA pathogenesis remain incompletely understood. This study investigates the regulatory networks underlying SSC dysfunction in NOA patients.

**Results:**

Using single-cell RNA sequencing, we identified significant downregulation of RGS14 in SSCs of NOA patients compared to normal testes. Immunofluorescence validation confirmed RGS14 localization primarily in SSCs. Functional assays demonstrated that RGS14 knockdown in SSC lines markedly suppressed cell proliferation and induced apoptosis. RNA-sequencing analyses revealed that RGS14 deficiency inhibited PLPP2 expression and MAPK signaling activation. Notably, PLPP2 overexpression rescued the phenotypic defects caused by RGS14 depletion. Protein-protein interaction assays and co-immunoprecipitation experiments further established that RGS14 physically interacts with GNAI3 to coordinately regulate cell proliferation and PLPP2 expression. Expression validation in NOA testes demonstrated concurrent downregulation of GNAI3 and PLPP2 in NOA patients, implicating their dysregulation in spermatogenic failure.

**Conclusion:**

Our findings uncover a novel RGS14-GNAI3-PLPP2 regulatory axis critical for SSC homeostasis. The dysregulation of these molecules contributes to SSC dysfunction and NOA pathogenesis. These data not only elucidate RGS14's role in SSC fate determination but also identify RGS14 and its interactome as promising therapeutic targets for restoring spermatogenesis in male infertility.

## Introduction

Reproductive health is a fundamental human right and a critical component of societal development. However, the infertility rate has been steadily increasing due to environmental pollution, high levels of urban living pressure, delayed marriage and childbearing age, and other factors. By 2020, the infertility rate had reached nearly 18%, with approximately 50% of cases attributed to male factors (Agarwal et al., 2021; Qiao et al., 2021). A significant decline in sperm quantity and concentration, as well as genetic mutations leading to sperm developmental disorders, are key contributors to infertility (Levine et al., 2023; Virtanen et al., 2017).

Spermatogonial stem cells (SSCs) are essential for continuous sperm production and the maintenance of male fertility (Di Persio and Neuhaus, 2023). They possess the unique abilities of self-maintenance, renewal, and differentiation (Brinster and Zimmermann, 1994; Gul et al., 2020). Understanding the mechanisms of their development and differentiation is crucial for clinically activating and utilizing SSCs to treat male infertility. Current research on SSCs has primarily focused on the maintenance and self-renewal mechanisms in adult mouse testes. The PI3K/AKT and Src pathways are recognized as core signaling pathways involved in SSC self-renewal (Kanatsu-Shinohara and Shinohara, 2013). Glial cell line-derived neurotrophic factor (GDNF), secreted by Sertoli cells, activates these pathways through the receptors GFRA1 and c-Ret within SSCs (Meng et al., 2000), initiating a series of genes and transcription factors related to SSC maintenance and self-renewal, such as *Etv5*, *Bcl6b*, and *Lhx1* (Ishii et al., 2012; Makela and Hobbs, 2019). Fibroblast growth factor (FGF), also secreted by Sertoli cells, not only activates the Src pathway but also synergizes with colony stimulating factor 1 (CSF1), secreted by interstitial cells, to regulate SSCs in conjunction with GDNF(Masaki et al., 2018; Potter and DeFalco, 2017). Additionally, promyelocytic leukemia zinc finger protein (PLZF) is a crucial transcription factor necessary for SSC maintenance and self-renewal, promoting these processes by relieving mTORC1 pathway inhibition of the GDNF receptors GFRA1 and c-Ret, while SALL4 can antagonize the function of PLZF (Buaas et al., 2004; Hobbs et al., 2010; Lovelace et al., 2016).

Despite these insights, most studies have been conducted in mice. The cell types and biochemical phenotypes of human spermatogonial stem cells differ from those of rodent stem cells (Hermann et al., 2018; Muciaccia et al., 2013), suggesting that the molecular regulatory mechanisms governing the fate determination of human and rodent spermatogonial stem cells may vary. Several studies have begun to explore human SSC proliferation, self-renewal, and apoptosis. For instance, microRNA-1908-3p promotes SSC proliferation by degrading KLF2 (Chen et al., 2020), and FGF5 stimulates human SSC proliferation through the activation of AKT and ERK (Tian et al., 2019). Our previous work has shown that ASB9 (Li et al., 2023), TCF3 (Zhou et al., 2021), MAGEB2 (Zhao et al., 2023), SPOCD1 (Zhou et al., 2022) and PTN (Zhao et al., 2024) are specifically expressed in human SSCs and regulate their self-renewal, proliferation, and apoptosis. However, the fate determination of human spermatogonial stem cells and the molecular mechanisms underlying spermatogenic disorders remain incompletely understood.

Regulator of G-protein signaling 14 (RGS14) is a multifunctional protein that integrates the G protein and H-Ras signaling pathways (Shu et al., 2010). It contains an RGS domain that binds to active Gαi/o-GTP subunits, promoting GTP hydrolysis, and a G protein regulatory (GPR) motif that selectively binds inactive Gαi1/3-GDP subunits, forming a stable heterodimer at cellular membranes (Vellano et al., 2013). RGS14 also includes two tandem Ras/Rap-binding domains (RBDs) that interact with H-Ras, preferentially binding activated H-Ras-GTP in live cells to increase H-Ras cellular activity (Hollinger et al., 2001). This interaction is regulated by inactive Gαi1-GDP and G protein-coupled receptors (GPCRs), highlighting RGS14’s role as a key regulator of signal transduction, particularly in hippocampal-based learning and memory (Lee et al., 2010). However, its role in SSC fate determination and spermatogenesis remains unclear.

In this study, we analyzed the scRNA-seq profiles of NOA and normal testes, revealing a significant reduction in SSCs in NOA and a marked downregulation of the RGS14 gene in these cells. Knockdown of RGS14 in a human SSC line notably inhibited cell proliferation and downregulated the expression of proteins associated with self-renewal while increasing apoptosis. RNA sequencing revealed a significant decrease in PLPP2 gene expression following RGS14 knockdown, and overexpression of PLPP2 mitigated the cellular phenotypic defects induced by RGS14 downregulation. Through database predictions and experiments such as protein immunoprecipitation, GNAI3 was confirmed to be a molecular partner in RGS14-mediated regulation of SSC function. Additionally, we observed significant downregulation of both PLPP2 and GNAI3 in NOA testes. These findings provide novel insights into the molecular mechanisms underlying SSC dysfunction in NOA and potential therapeutic targets for male infertility.

## Materials and methods

### Ethical statement and sample collection

The study was approved by the ethics committee of Hunan Normal University (No. 2024596), and all participants provided signed informed consent. Testicular tissues were collected from 15 patients aged 25–46 years who underwent testicular biopsy, with approximately 25 mg of tissue from each patient. To eliminate blood cells, the samples were thoroughly rinsed with sterile PBS on at least three occasions. The samples were subsequently preserved in liquid nitrogen or treated with 4% PFA or Bouin’s fixative solution.

### scRNA-seq analysis of normal and NOA testes

Single-cell sequencing data were analyzed primarily via the Seurat 4 R package (https://github.com/satijalab/Seurat) (Hao et al., 2021). The Read10x function was used to import the scRNA-seq datasets GSE149512 (Zhao et al., 2020) (3 NOA testicular samples) and GSE112013 (Guo et al., 2018) (3 normal testicular samples) into R, generating the Seurat object. Gene expression data were then filtered, retaining cells with gene expression values ranging from 500–7,500 and less than 20% of genes related to mitochondria. All the mitochondrial and ribosomal genes were removed on the basis of their nomenclature. Duplicate entries were detected and eliminated via the DoubletFinder R package (https://github.com/chris-mcginnis-ucsf/DoubletFinder) (Stoeckius et al., 2018). The NormalizeData and FindVariableFeatures functions were applied to each Seurat object. All the Seurat objects were combined via the FindIntegrationAnchors and IntegrateData functions. Data clustering was performed after the default UMAP technique was used, and cell types were subsequently determined by evaluating the expression of cellular markers. The plot1 cell R package (https://github.com/HumphreysLab/plot1 cell) was used to plot graphs after identifying and clustering the cells (Wu et al., 2022). Transcriptional data of SSCs were analyzed via the clusterProfiler R package (https://github.com/YuLab-SMU/clusterProfiler) for differentially expressed genes and Gene Ontology (GO) analysis (Wu et al., 2021). To investigate the expression of RGS14 during SSC development, data from SSCs were collected, reclustered via Seurat, and then imported into the Monocle3 R package (https://cole-trapnell-lab.github.io/monocle3/) to create developmental trajectories for SSCs(Cao et al., 2019). All the dot, line, and violin plots were created and modified via ggplot2 (https://github.com/tidyverse/ggplot2) in R (Ginestet, 2011).

### Culture of human SSC lines

The human SSC line was established by introducing the large T antigen into GPR125-positive undifferentiated spermatogonia from humans (Hou et al., 2015). This human SSC line retains several characteristics and markers of primary SSCs, including GFRA1, RET, and PLZF, but does not express testicular endosomal cell markers such as SOX9. The immortalized human SSCs were cultured at 34°C with a 5% CO_2_ concentration in DMEM/F12 (Gibco, Carlsbad, CA, United States) supplemented with 10% FBS (Gibco). The cells were subcultured every 48–72 h with 0.5 g per liter of trypsin and 0.53 mmol per liter of EDTA from Invitrogen.

### The process of extracting total RNA, performing reverse transcription PCR, and conducting quantitative PCR

Following the manufacturer’s instructions, we extracted total RNA from isolated cells using RNAiso Plus reagent (Takara, Tokyo, Japan). The quality and concentration of the extracted RNA were evaluated using a Nanodrop spectrophotometer from Thermo Fisher Scientific. Commercial kits (Roche, Basel, Switzerland) were used for the reverse transcription of cDNA. In accordance with the manufacturer’s instructions, we performed qPCR using the ABI Prism 7,700 system from Applied Biosystems. To determine the relative levels of mRNA, we employed the 2^−ΔΔCT^ method, with β-actin serving as an internal reference. After thoroughly analyzing each sample, we conducted three replicates and calculated the average results. All primers were obtained from PrimerBank (https://pga.mgh.harvard.edu/primerbank/), and their sequences are listed in Supplementary Table S1.

### Immunohistochemistry and immunofluorescence of tissue sections

The testicular sections were deparaffinized with xylene and rehydrated with graded ethanol for immunohistochemistry. Heat-induced antigen retrieval was then performed by immersing the samples in 0.01 mol/L sodium citrate buffer and heating them at 98°C for 18 min. After cooling and washing, the sections were incubated with 3% hydrogen peroxidase (Zsbio, Beijing, China) to block endogenous peroxidase activity. Following three rinses with PBS, the tissue sections were treated with 0.25% Triton X-100 (Sigma, St. Louis, MO, United States) for 15 min to increase their permeability. Nonspecific antigens were blocked by incubating the sections in 5% bovine serum albumin at room temperature for 1 hour. The sections were then incubated overnight at 4°C with the primary antibodies listed in Supplementary Table S2. After three rinses with PBS, the sections were treated with horseradish peroxidase-conjugated goat anti-rabbit secondary antibody and incubated at room temperature for 1 hour. Color development was achieved using the use of a 3,3′-diaminobenzidine chromogen kit (Dako, Glostrup, Denmark). The nuclei were stained with hematoxylin for 7 min at room temperature. For immunofluorescence, the primary antibody was incubated at 4°C for 16 h, followed by chromogenic development using an Alexa Fluor-conjugated secondary antibody. The cell nuclei were counterstained with DAPI. Microscopy images of the testicular sections were captured and analyzed via a Zeiss microscope (Carl Zeiss, Jena, Germany).

### Protein extraction, western blotting and co-immunoprecipitation

Testicular tissue and cells were lysed via RIPA buffer (Thermo Fisher Scientific, Waltham, MA, United States) on ice for 15 min. After centrifugation at 12,000 × g for 15 min, the supernatants were collected for total protein extraction and Western blot analysis. The overall protein concentration was determined using the BCA Kit according to the manufacturer’s instructions. Each sample was analyzed using sodium dodecyl sulfate‒polyacrylamide gel electrophoresis and Western blot analysis, following a previously described method, with 20 μg of total protein. The antibodies used are listed in Supplementary Table S2. To visualize the protein bands, a chromogenic solution with enhanced chemiluminescence (Thermo Fisher Scientific) was used, and the resulting chemiluminescent signals were captured and analyzed via Fusion FX (Vilber Lourmat). For the co-immunoprecipitation assay, cell lysates were prepared using RIPA buffer supplemented with protease and phosphatase inhibitors. The protein concentration was determined using the Bradford assay. Equal amounts of protein were incubated with specific antibodies against the target proteins overnight at 4°C with gentle rotation. Subsequently, protein A/G magnetic beads were added and incubated for 2 h at 4°C. The immune complexes were washed, eluted, and analyzed by Western blotting to detect the interacting proteins. All the samples were analyzed three times, and the average results were calculated.

### siRNA and plasmid transfection

Zorin (Shanghai, China) designed and synthesized RGS14 siRNAs, while PLPP2 overexpression plasmids were prepared by SinoBiological (Beijing, China). The immortalized human SSCs were transfected with either 100 nmol/L of siRNAs or 2.5 μg of plasmids using Lipofectamine 3,000 (Life Technologies) following the manufacturer’s instructions. The cells were collected 48 h post-transfection to extract protein and RNA for PCR and Western blot analysis.

### Cell viability assay

A CCK-8 Kit (Dojindo, Kumamoto, Japan) was used to assess the viability of the SSCs, adhering strictly to the protocols stipulated by the manufacturer. Next, the cells were subjected to a three-hour incubation period in culture medium enriched with 100 mL/L CCK-8 reagents. The absorbance at 450 nm was subsequently determined using a microplate reader from Thermo Fisher Scientific.

### EdU incorporation assay

An EdU labeling kit (RiboBio, Guangzhou, China) was used to detect DNA synthesis. Human SSCs were seeded into 96-well plates at a density of 5,000 cells per well in culture medium supplemented with 50 μmol/L EdU. Following a 12 h incubation, the cells were washed with DMEM and fixed with 40 g/L PFA. Glycine (2 mg/mL) neutralized the cells, which were then permeabilized with 5 mL/L Triton X-100 for 10 min at room temperature. The Apollo staining reaction buffer was used to detect EdU, and the cell nuclei were stained with DAPI. Microscopy images of the EdU-positive cells were captured and analyzed using a Zeiss fluorescence microscope. A minimum of 500 cells were evaluated in each sample.

### Cell apoptosis assay

Following 48 h of transfection with siRNA, the cells were subjected to trypsin/EDTA treatment and subsequently rinsed twice with ice-cold PBS. A minimum of 10^6 cells were then resuspended in Annexin V binding buffer (BD Biosciences, San Jose, CA, United States of America) and incubated with 5 µL of APC-labeled Annexin V for 15 min at room temperature. The cells were subsequently treated with 10 µL of PI and incubated for an additional 10 min prior to the assay. The degree of cell apoptosis was assessed via a BD Biosciences C6 flow cytometer.

An *In Situ* Cell Death Detection Kit (Roche) was used to examine the influence of plasmids on the apoptosis of the human SSC line. The cells were treated with proteinase K (20 mg/mL) for 15 min at room temperature and then incubated for 1 hour with dUTP labeling/terminal deoxynucleotidyl transferase (TdT) enzyme buffer in the absence of light. The cell nuclei were counterstained with DAPI. At least 500 cells per sample were analyzed via a Zeiss fluorescence microscope.

### RNA-seq

Total RNA from cells was isolated via a TRIzol reagent kit (Invitrogen, Carlsbad, CA, United States). Personalbio (Shanghai, China) conducted RNA sequencing and preliminary analysis, with the detailed procedures outlined in our prior research (Li et al., 2023). The ClusterGVis R package (https://github.com/junjunlab/ClusterGVis) facilitated trend and Gene Ontology (GO) enrichment analyses, whereas the ClusterProfiler R package enabled Kyoto Encyclopedia of Genes and Genomes (KEGG) enrichment analysis. Finally, the plot1 cell R package was used to generate dot plots.

### Statistical analysis

The R programming language employed the dplyr package for data analyses (https://dplyr.tidyverse.org) (Wickham and François, 2014). Each experiment was replicated at least three times. The data are presented as the means ± standard deviations. Variances among groups were evaluated using a *t*-test. A significance level of <0.05 indicated statistical significance.

## Results

### Single-cell transcriptomic atlas of normal and NOA testes

**FIGURE 1:**
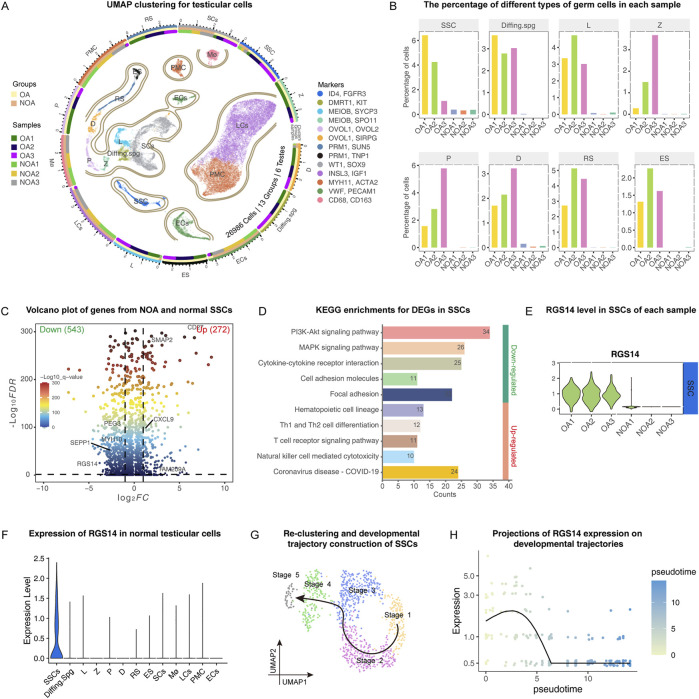
Single-cell transcriptome analysis of normal and NOA testicular samples. **(A)** UMAP clustering of normal and NOA testicular cells. The 26,986 cells from six samples were divided into 13 clusters, each cluster was colored sequentially, and the marker used for cell identification is shown on the right side of the image. The outermost ring symbolizes distinct clusters; the middle ring indicates the proportion of different groups within each cluster; and the inner ring represents the percentage of individual samples in each cluster. The groups and samples are shown on the left side of the image. **(B)** The bar graphs show the proportion of germ cells in each sample, including the following: SSC, Diffing. spg, L, Z, P, D, RS and ES. **(C)** Volcano plot demonstrating the gene distribution of SSCs in NOA and normal testicular samples. The right side of the vertical dotted line represents significantly upregulated genes. The left side of the vertical dashed line represents significantly downregulated genes. **(D)** KEGG analysis of genes that were differentially expressed between the NOA and normal samples of SSCs. The numbers in the bar graph represent the counts enriched in this signaling pathway. **(E)** Violin plots demonstrating the expression of RGS14 in the SSCs of each sample. **(F)** Violin plot demonstrating the expression of RGS14 in normal testicular cells. **(G)** Reclustering of SSCs in the normal testis. The SSC was reclassified into five different stages, and the arrows represent the developmental direction of the SSCs. **(H)** Expression levels of RGS14 along the SSC developmental trajectory. SSC: spermatogonial stem cell, Diffing. spg: differentiating spermatogonia, L: leptotene spermatocyte, Z: zygotene spermatocyte, P: pachytene spermatocyte, D: diplotene spermatocyte, RS: round spermatid.

To investigate the developmental process of SSCs and their role in NOA, we reintegrated and analyzed scRNA data from three NOA cases (GSE149512) (Zhao et al., 2020) and three normal testis samples (GSE119013) (Guo et al., 2018). After eliminating low-quality cells, we categorized 29,686 cells from six testes into 13 distinct clusters through UMAP clustering. These clusters encompass various cell types: spermatogonial stem cell (SSC), Differentiating spermatogonia (Diffing.spg), leptotene spermatocytes (L), zygotene spermatocytes (Z), pachytene spermatocytes (P), diplotene spermatocytes (D), round spermatids (RS), elongating spermatids (ES), Sertoli cells (SC), Leydig cells (LC), peritubular myoid cells (PMC), epithelial cells (EC) and macrophages (Mø). The markers associated with these clusters are illustrated in Figure 1A and include *ID4*, *KIT*, *MEIOB*, *SPO11*, *OVOL2*, *SIRPG*, *SUN5*, *PRM1*, *WT1*, *INSL3*, *MYH11*, *VWF*, and *CD68*. Figure 1A depicts three concentric rings: the outermost ring symbolizes distinct clusters; the middle ring indicates the proportion of different groups within each cluster; and the inner ring represents the percentage of individual samples in each cluster. Upon quantifying germ cells in each sample, we observed a significant reduction in all germ cells within the NOA samples. Notably, the SSCs, which were our primary focus, also exhibited a substantial decrease (Figure 1B). We subsequently extracted the data pertaining to all the SSCs and conducted a more in-depth analysis to identify the DEGs and associated signaling pathways. Our findings indicated that, in NOA samples, 534 genes were significantly downregulated, whereas 272 genes were significantly upregulated (Figure 1C). The majority of the downregulated genes were predominantly involved in the AKT and MAPK signaling pathways (Figure 1D), which are known to play crucial roles in SSC proliferation and self-renewal. Within the group of downregulated genes, *RGS14*, which displayed a notable reduction across all NOA samples, attracted particular attention. These findings suggest that RGS14 might play a critical role in the process of spermatogenesis (Figure 1E). A more thorough analysis revealed that *RGS14* is predominantly localized in normal SSC samples (Figure 1F). By utilizing Monocle3, these SSCs can be classified into five distinct subgroups. Following this classification, we designated these subgroups sequentially from stage 1 to stage 5, according to their developmental progress (Figure 1G). Importantly, as development progresses, the expression level of *RGS14* consistently decreases (Figure 1H). These results indicate that *RGS14* is localized primarily in SSCs and that its expression is downregulated in NOA, potentially contributing to the dysregulation of spermatogenesis.

### The expression of RGS14 in normal and NOA testicular tissues

**FIGURE 2:**
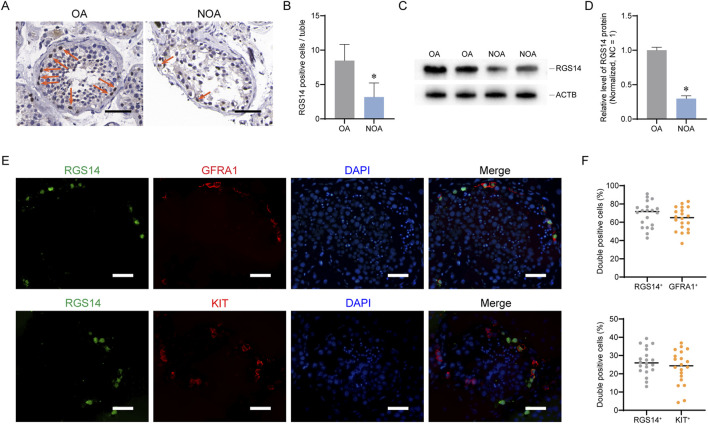
Expression and distribution of RGS14 in NOA and normal testicular tissues. **(A)** Immunohistochemistry was used to detect the distribution of RGS14 in NOA and normal testes. The arrows represent RGS14-positive cells. Scale bar, 100 μm. **(B)** Bar graph demonstrating the number of RGS14-positive cells in NOA seminiferous tubules and normal seminiferous tubules. RGS14-positive cells were significantly reduced in NOA testes. **(C)** Western blot analysis of the overall levels of RGS14 in NOA and normal testes. **(D)** Bar graph demonstrating the overall levels of RGS14 in NOA and normal testicular tissues. RGS14 protein levels were significantly reduced in NOA testes. **(E)** Immunofluorescence detection of RGS14 localization in spermatogonia subpopulations. The green signal is RGS14, the red signals are GFRA1 (SSC marker) and KIT (differentiated spermatogonia marker), and the blue signal is DAPI. Scale bar, 50 μm. **(F)** Dot plots showing the percentage of RGS14 colocalized with GFRA1 and KIT. Each dot represents a counting result. * represents *P* < 0.05.

To validate the results of the scRNA-seq analysis, we examined the distribution of RGS14 in OA (normal spermatogenesis) and NOA via immunohistochemistry. The number of RGS14-positive cells was significantly reduced in the NOA samples (n = 50, mean ± SD: 8.48 ± 2.35 vs. 3.18 ± 2.06; *P* < 0.05, *t*-test) (Figures 2A,B). The Western blot data also revealed a significant reduction in the overall level of RGS14 protein in the NOA (n = 3, RGS14 protein, mean ± SD: 1.00 ± 0.04 vs. 0.29 ± 0.04; *P* < 0.05, t-test) (Figures 2C,D). Additionally, we analyzed the localization of RGS14 in normal spermatogonia via immunofluorescence. The results indicated that approximately 63.73% ± 12.59% (n = 20) of the SSCs (GFRA1 positive) expressed RGS14, whereas only approximately 23.52% ± 9.33% (n = 20) of the differentiated spermatogonia (KIT positive) expressed RGS14 (Figures 2E,F). These findings are consistent with the bioinformatics results, which suggest that RGS14 is downregulated in NOA and predominantly expressed in spermatogonial stem cells.

### The effects of RGS14 on SSC proliferation and apoptosis

**FIGURE 3:**
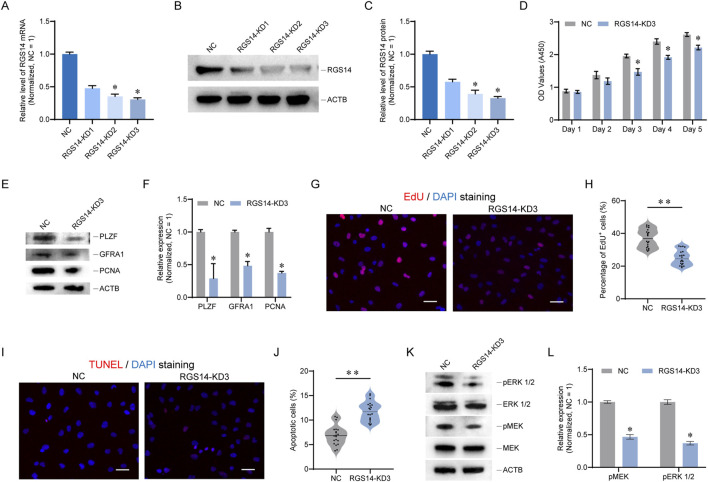
RGS14 knockdown inhibits SSC proliferation and induces apoptosis. **(A)** Bar graph showing the mRNA levels of RGS14 after RGS14 knockdown. **(B)** Western blot detection of the protein levels of RGS14 after RGS14 knockdown. **(C)** Bar graph showing the protein levels of RGS14 after RGS14 knockdown. **(D)** CCK8 was used to detect cell proliferation from day 1 to day 5 after RGS14-KD3 transfection. **(E)** Western blot analysis of the expression levels of SSC self-renewal-related proteins. These genes include PLZF, GFRA1 and PCNA. **(F)** Bar graph showing the protein levels of PLZF, GFRA1 and PCNA in **(E) (G)** EdU was used to detect cellular DNA synthesis. EdU signals are shown in red, and DAPI signals are shown in blue. Scale bar, 20 μm. **(H)** Violin plot showing the proportion of EdU-positive cells in **(G)**. Each dot represents one count. **(I)** TUNEL staining was used to detect cell apoptosis. The TUNEL signal is shown in red, and DAPI is shown in blue. Scale bar, 20 μm. **(J)** Violin plot showing the proportion of TUNEL-positive cells in **(I)**. Each dot represents one count. **(K)** Western blotting was used to detect the phosphorylation of MEK and ERK 1/2 proteins. **(L)** Bar graph showing the protein levels of phosphorylated MEK and ERK 1/2 in **(K)**. * represents *P* < 0.05. ** represents *P* < 0.01.

To investigate the regulatory effects of RGS14 on human SSCs, a human SSC line was utilized. Using siRNA, we knocked down RGS14 in the SSC line and observed that RGS14-KD3 had the best inhibitory effect, as evidenced by both qPCR and Western blot assays (n = 3, RGS14 protein, mean ± SD: 1.00 ± 0.05 vs. 0.33 ± 0.02; *P* < 0.05, t-test) (Figures 3A–C). Following the knockdown of RGS14, we examined cell proliferation via a CCK8 assay and found that it was significantly reduced from the third to the fifth day after RGS14-KD3 transfection (Figure 3D). We also examined the expression of PLZF, GFRA1, and PCNA, which are proteins related to SSC self-renewal, and found that their overall levels were significantly reduced (n = 3; *t*-test, *P* < 0.05) (Figures 3E,F). The results of an EdU assay indicated that the reduction in RGS14 resulted in attenuated DNA synthesis (Figures 3G,H). However, the TUNEL assay results revealed an increase in DNA breaks and a significant increase in the overall percentage of apoptotic cells (Figures 3I,J). RGS14 is implicated in the regulation of MAPK signaling, a pathway that contributes to SSC proliferation and self-renewal (Kanatsu-Shinohara and Shinohara, 2013; Vellano et al., 2011). Consequently, we investigated the phosphorylation status of MEK and ERK 1/2, which are pivotal molecules in the MAPK signaling cascade. The results revealed that RGS14 downregulation inhibited the phosphorylation of both ERK1/2 and MEK, suggesting that MAPK signaling was attenuated (n = 3; *t*-test, *P* < 0.05) (Figures 3K,L). These results suggest that knockdown of RGS14 leads to a significant reduction in proliferation and promotes apoptosis in SSC lines.

### Downstream target screening of RGS14 via RNA sequencing

**FIGURE 4:**
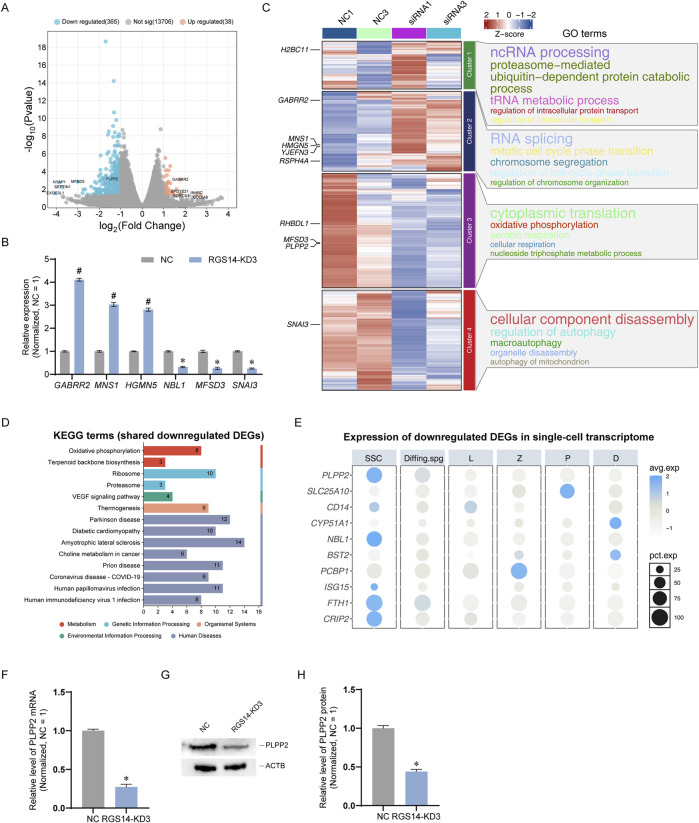
RNA sequencing analysis of downstream genes and enriched pathways of RGS14. **(A)** Volcano plot showing the distribution of all genes identified by RNA sequencing. Genes significantly upregulated after RGS14 knockdown are shown in orange, whereas genes significantly downregulated are shown in blue. **(B)** qPCR was used to verify six randomly selected DEGs according to the RNA sequencing results. **(C)** Heatmap showing the expression trends of all identified genes. The scaled gene expression levels are colored according to the Z score at the upper right. All genes were categorized into 4 clusters, and genes in cluster 2 were significantly upregulated after RGS14 knockdown, whereas genes in clusters 3 and 4 were significantly downregulated. The top 10 DEGs are labeled. Right: GO enrichment of genes in each cluster; the top five terms are shown. **(D)** KEGG enrichment analysis of the top 100 significantly downregulated genes. The numbers in the bars represent the number of downregulated genes that were enriched with that KEGG term. **(E)** Dot plots showing the expression projections of selected genes in Figure 1A single-cell transcriptome. The scaled gene expression levels are colored according to the Z score at right. The size of the dot represents the percentage of cells expressing that gene. **(F)** The mRNA level of PLPP2 after RGS14 knockdown was detected by qPCR. **(G)** Western blot detection of the PLPP2 protein after RGS14 knockdown. **(H)** Bar graph showing the protein levels of PLPP2 after RGS14 knockdown. # Represents a significant upregulation compared with the NC group, and *P* < 0.05. * Represents a significant downregulation compared with the NC group, and *P* < 0.05.

To elucidate the downstream targets of RGS14, we conducted RNA sequencing on cells 48 h post transfection. After filtering out genes with low expression and unidentifiable sequences, we identified a total of 14,109 genes. Among these genes, 365 genes were significantly downregulated, 38 were significantly upregulated, and 13,706 genes exhibited no significant changes (Supplementary Table S3). The distribution of all genes is depicted in the volcano plot shown in Figure 4A. To confirm the RNA sequencing results, we randomly selected six DEGs for further validation via qPCR. The findings revealed that *GABRR2*, *MNS1*, and *HMGN5* were significantly upregulated, whereas *NBL1*, *MFSD3*, and *SNAI3* were significantly downregulated, which aligns with the RNA sequencing data (Figure 4B). On the basis of the gene expression data from each sample set, we performed expression trend analysis. All the genes were categorized into four clusters; cluster 2 primarily contained genes whose expression tended to increase, whereas clusters 3 and 4 predominantly consisted of genes whose expression tended to decrease. We conducted GO enrichment analysis on the genes within each cluster and discovered that processes such as RNA splicing were upregulated, whereas processes such as cytoplasmic translation and autophagy were significantly downregulated (Figure 4C). Additionally, we performed Kyoto Encyclopedia of Genes and Genomes (KEGG) enrichment analysis on the significantly downregulated genes, revealing that pathways such as oxidative phosphorylation was significantly downregulated. This finding was consistent with the GO enrichment results from the trend analysis (Figure 4D). Furthermore, we screened certain genes potentially associated with SSC proliferation and apoptosis, including genes such as *PLPP2* (Wang et al., 2023), *SLC25A10* (Wang et al., 2020), and *CD14* (Cheah et al., 2015), and examined their expression in normal testes via scRNA-seq data. Notably, we found that *PLPP2*, *NBL1*, *FTH1*, and *CRIP2* were primarily localized in SSCs (Figure 4E). We subsequently confirmed that the genes localized to SSCs were significantly downregulated at both the mRNA and protein levels. The results indicated that PLPP2 was significantly downregulated at both the mRNA and protein levels (n = 3; *t*-test, *P* < 0.05) (Figures 4F–H), whereas no significant changes in other genes at the protein level were detected (data not shown). These findings suggest that RGS14 knockdown leads to significant downregulation of genes such as *PLPP2* and impacts signaling pathways such as oxidative phosphorylation.

### PLPP2 alleviates phenotypic defects caused by RGS14 knockdown

**FIGURE 5:**
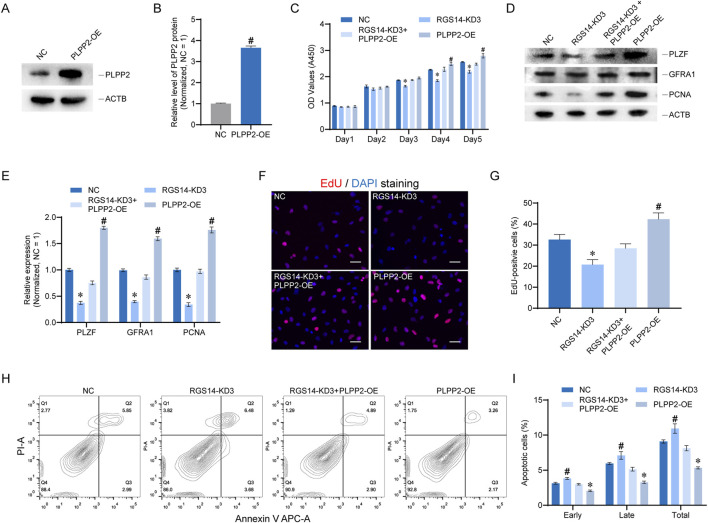
PLPP2 alleviates phenotypic changes caused by RGS14 knockdown in SSC lines. **(A)** Western blot analysis of protein expression after PLPP2 overexpression. **(B)** Bar graph showing protein levels after PLPP2 overexpression. **(C)** CCK8 was used to detect cell proliferation in the NC, RGS14-KD3, RGS14-KD3+PLPP2-OE, and PLPP2-OE groups from 1 to 5 days after transfection. **(D)** Western blotting was performed to detect the expression of genes related to SSC proliferation and self-renewal in these four groups. These genes include PLZF, GFRA1 and PCNA. **(E)** Bar plot showing the level of each protein relative to the NC group in **(D)**. **(F)** EdU was used to detect DNA synthesis in these four groups. The red color represents EdU positivity, and the nuclei were counterstained with DAPI. Scale bar, 20 μm. **(G)** Bar plot demonstrating the proportion of EdU-positive cells relative to that in the NC group in **(F)**. **(H)** Flow cytometry was used to detect the proportion of apoptotic cells in the four groups. **(I)** Bar plots showing the proportion of apoptotic cells in each group relative to that in the NC group. These include early apoptosis, late apoptosis and total apoptosis. # Represents a significant upregulation compared with the NC group, and *P* < 0.05. * Represents a significant downregulation compared with the NC group, and *P* < 0.05.

To elucidate the role of PLPP2 in RGS14-mediated SSC proliferation and apoptosis, we conducted phenotypic rescue experiments. We engineered a plasmid for PLPP2 overexpression (PLPP2-OE) and validated its efficacy via Western blot analysis. The data revealed that the PLPP2-OE plasmid significantly elevated PLPP2 protein expression post-transfection (n = 3; *t*-test, *P* < 0.05) (Figures 5A,B). We subsequently co-transfected both RGS14-KD3 and PLPP2-OE cells to assess cell proliferation and apoptosis. CCK8 assays revealed that PLPP2 overexpression enhanced cell proliferation on the fourth- and fifth-days post-transfection, whereas concurrent transfection of RGS14-KD3 and PLPP2-OE mitigated the decrease in cell proliferation induced by RGS14 knockdown (Figure 5C). The expression levels of proteins involved in SSC proliferation and self-renewal, including PLZF, GFRA1, and PCNA, were also restored upon PLPP2 overexpression (n = 3; *t*-test, *P* < 0.05) (Figures 5D,E). Comparable outcomes were observed in the EdU assays, where PLPP2 overexpression notably augmented cellular DNA synthesis and counteracted the phenotypic anomalies associated with RGS14 knockdown (n = 3; *t*-test, *P* < 0.05) (Figures 5F,G). Moreover, apoptosis detection via flow cytometry demonstrated that PLPP2 also reversed the changes in apoptosis triggered by RGS14 (n = 3; *t*-test, *P* < 0.05) (Figures 5H,I). Collectively, these findings suggest that PLPP2 overexpression ameliorates the phenotypic deficits induced by RGS14 knockdown, suggesting that PLPP2 is a downstream target of RGS14.

### RGS14 interacts with GNAI3 and affects SSC proliferation

**FIGURE 6:**
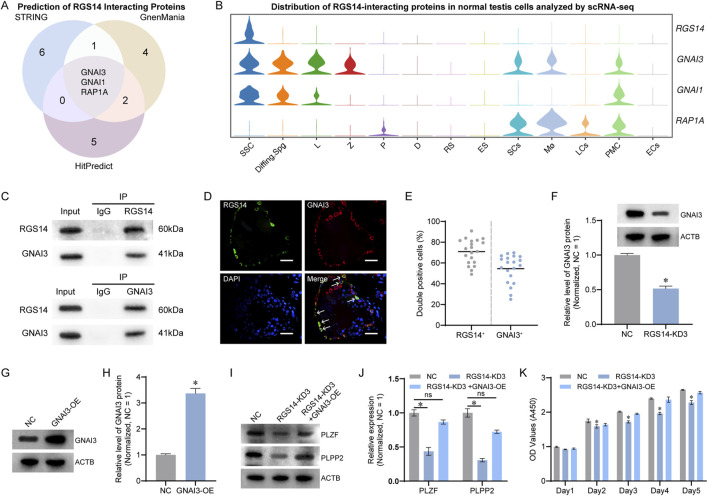
Prediction and validation of RGS14-interacting proteins in human SSCs. **(A)** Venn diagram showing the results of the STRING, GeneMania and HitPredict databases in predicting RGS14-interacting proteins, with GNAI3, GNAI1 and RAP1A as the intersections of the predictions. **(B)** Violin plots demonstrating the expression of GNAI3, GNAI1, and RAPA1 in normal human testis scRNA-seq profiles. RGS14, GNAI3, and GNAI1 are robustly expressed in SSCs, whereas RAPA1 is expressed predominantly in somatic cells. **(C)** Co-IP verification of the interaction between RGS14 and GNAI3. Western blot analysis revealed a clear GNAI3 band after enrichment with an RGS14 antibody. A clear RGS14 band was also detected after enrichment with the anti-GNAI3 antibody. **(D)** Immunofluorescence detection of RGS14 and GNAI3 in normal testes. RGS14 (green) and GNAI3 (red) are abundantly colocalized in cells near the seminiferous tubules. The white arrows indicate co-expressed cells. Scale bar, 50 μm. **(E)** The dot plot showing the proportion of co-expressed cells in **(D)**. Each dot represents a counting result. **(F)** Western blot analysis revealed downregulation of GNAI3 expression after RGS14 knockdown. **(G)** Western blot analysis of protein levels after GNAI3 overexpression. **(H)** The bar graph shows the relative protein levels of GNAI3 in **(G)**. **(I)** Western blot analysis of the levels of PLZF and PLPP2 in the NC, RGS14-KD3 and RGS14-KD3+GNAI3-OE groups. Both PLZF and PLPP2 were significantly downregulated upon RGS14 knockdown, whereas overexpression of GNAI3 alleviated the downregulation of protein levels caused by RGS14 deficiency. **(J)** The bar graph shows the relative levels of PLPP2 and PLZF in **(I)**. **(K)** CCK8 assay for cell proliferation in all three groups. The overexpression of GNAI3 partially rescued the downregulation of cell proliferation caused by RGS14 knockdown. * indicates *P* < 0.05. KD: knockdown. OE: Overexpression. ns: not significant.

RGS14, characterized as a scaffold protein, orchestrates intracellular signaling pathways. Using the STRING, GeneMania, and HitPredict databases, we predicted potential interaction partners of RGS14 and identified GNAI3, GNAI1, and RAP1A as candidates through an intersection of the prediction results (Figure 6A). Subsequent analysis of the scRNA-seq landscape revealed robust expression of RGS14, GNAI3, and GNAI1 in SSCs, whereas RAP1A was virtually absent, diminishing its ability to interact with RGS14 in SSCs (Figure 6B). Co-immunoprecipitation assays confirmed significant interactions between RGS14 and GNAI3 (Figure 6C), with negligible evidence of interaction with GNAI1 (data not shown). The immunofluorescence results further demonstrated substantial colocalization of RGS14 and GNAI3 in the testes, approximately 70.94% ± 11.90% (n = 20) of RGS14-positive cells expressed GNAI3 (Figures 6D,E). Additionally, the knockdown of RGS14 coincided with the downregulation of GNAI3 expression (n = 3; *t*-test, *P* < 0.05) (Figure 6F). We then examined the role of GNAI3 in RGS14-mediated SSC proliferation. The overexpression of GNAI3 in the SSC lines partially restored the protein expression of PLZF and PLPP2, which was diminished upon RGS14 knockdown (n = 3; *t*-test, *P* < 0.05) (Figures 6G–J). CCK8 assays also revealed that GNAI3 overexpression mitigated the decrease in cell proliferation triggered by RGS14 knockdown (n = 3; *t*-test, *P* < 0.05) (Figure 6K). Collectively, these findings underscore GNAI3 as a molecular partner in RGS14-mediated regulation of SSC function.

### PLPP2 and GNAI3 expression was downregulated in NOA testes

**FIGURE 7:**
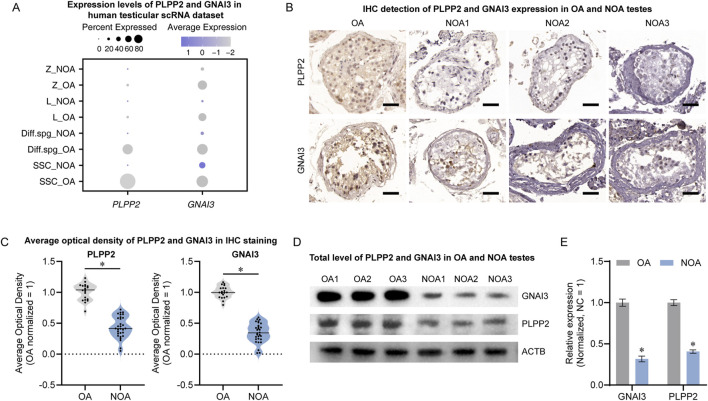
PLPP2 and GNAI3 levels in normal and NOA testes. **(A)** Distribution of PLPP2 and GNAI3 expression in human testicular scRNA-seq profiles. Both PLPP2 and GNAI3 were significantly downregulated in NOA testes. **(B)** Immunohistochemical detection of PLPP2 and GNAI3 expression in OA and NOA testes. The number of positive cells in NOA testes was significantly reduced. Scale bar, 50 μm. **(C)** Mean optical density analysis after PLPP2 and GNAI3 staining in **(B)**. The mean optical density was significantly lower in the NOA samples. **(D)** Western blot detection of PLPP2 and GNAI3 levels in OA and NOA testes. **(E)** Bar graphs showing the relative levels of PLPP2 and GNAI3. The expression of PIPPL2 and GNAI3 was significantly reduced in NOA testes. * indicates *P* < 0.05.

To explore the potential roles of PLPP2 and GNAI3 in NOA, we assessed their expression profiles in testes. In the scRNA-seq landscape, PLPP2 was primarily localized to SSCs, whereas GNAI3 exhibited a broader expression profile and was present in spermatogonia through early spermatocytes. Notably, both were downregulated in NOA testes (Figure 7A). The immunohistochemical results revealed a significant reduction in the number of PLPP2- and GNAI3-positive cells in the NOA, with a marked decrease in the mean optical density (optical density: OA, n = 20; NOA, n = 30; *t*-test, *P* < 0.05) (Figures 7B,C). Concurrently, Western blot analysis of total protein levels revealed a pronounced decrease in the protein expression of both PLPP2 and GNAI3 in NOA testes (n = 3; *t*-test, *P* < 0.05) (Figures 7D,E). These findings suggest that the significant reduction in PLPP2 and GNAI3 and their dysregulation in conjunction with RGS14 may be involved in the pathogenesis of NOA.

## Discussion

NOA represents the most severe form of spermatogenesis disorder, affecting approximately 1% of the male population, primarily because of nongenetic factors (Piechka et al., 2024). The limited understanding of the etiology and progression of NOA has resulted in a scarcity of treatment options. SSCs are pivotal for spermatogenesis in adult males, as they initiate and sustain sperm production throughout life (de Rooij and Russell, 2000; Di Persio et al., 2017). In mouse models, genes such as *Plzf* (Buaas et al., 2004), *Foxo1* (Goertz et al., 2011), and *Dot1l* (Lin et al., 2022) have been identified as crucial regulators of SSC self-renewal and proliferation. Disruption of these genes leads to an inability to maintain SSCs, culminating in germ cell loss and a testicular phenotype analogous to human NOA. While no single SSC gene mutation directly linked to NOA has been identified, the results of mouse experiments suggest that dysfunction of numerous SSC genes can lead to Sertoli cell-only syndrome (SCOS), a severe manifestation of NOA (Kanatsu-Shinohara et al., 2014). These findings underscore the significant role of SSCs in the development of NOA.

In our study, we analyzed single-cell transcriptional data from NOA and normal spermatogenesis testes. We found that a reduction in germ cell number occurred in almost all NOA samples, and further analysis of the differentially expressed genes from SSCs confirmed a significant reduction in RGS14. In fact, the number of spermatogonia was also drastically reduced, and our study did not explore spermatogonia or testicular somatic cell factors in NOA. In addition, many SSC genes, such as *PEG3* and *MYH10*, are downregulated in NOA, and many genes whose expression is upregulated remain to be further investigated.

RGS14, a multifunctional scaffold protein that integrates the G protein and H-Ras/MAPK signaling pathways, is enriched in CA2 hippocampal neurons in the brain (Vellano et al., 2011). This is where most of the previous research on RGS14 originated. The hippocampus is important for spatial learning and memory; however, RGS14 appears to be a negative regulator that may inhibit synaptic plasticity through MAPK signaling (Shu et al., 2010). RGS14 knockout (RGS14-KO) mice learn to navigate water mazes and locate underwater escape platforms faster, suggesting that the loss of RGS14 significantly improves the acquisition rate of spatial learning (Lee et al., 2010). However, the results of these studies appear to differ from our data. Our data suggest that RGS14 is predominantly distributed in male germline stem cells, whereas CA2 hippocampal neurons are terminally differentiated (Hollinger et al., 2001). Furthermore, RGS14 plays a negative regulatory role in hippocampal neurons (Lee et al., 2010), whereas it is significantly reduced in testes with dysgenic spermatogenesis, and its downregulation significantly inhibits SSC proliferation. Considering that RGS14 is a scaffolding protein, there may be differences in its intercalating proteins in stem cells and differentiated cells, leading to different roles of RGS14. Notably, RGS14 has been reported to interact with both GNAI1 and GNAI3 (Cho and Kehrl, 2007). However, our co-IP experiments revealed a significant interaction only with GNAI3. This discrepancy may arise from the low specificity of the antibodies used. We will subsequently investigate potential interactions via pull-down assays and the yeast two-hybrid system.

The formation and development of SSCs is a complex process that involves multiple developmental events and signaling pathways. Although the underlying mechanisms are still unclear, SSC formation may involve several stages, including the migration of PGCs, the differentiation of PGCs into prospermatogonia (proSg), and the transformation of prospermatogonia into SSCs(McCarrey, 2013). RGS14 has been reported to be expressed in zygotes and is required to complete the first mitotic division of the mouse embryo (Martin-McCaffrey et al., 2004). Our data suggest that in adult testes, RGS14 is predominantly expressed in SSCs. Whether RGS14 is expressed in PGCs and proSgs and influences the process of SSC formation at an earlier stage is not known. We will investigate this by analyzing data from embryonic testes and by constructing RGS14 knockout mice. Although we explored the downstream target genes of RGS14 via RNA-seq and confirmed the role of *PLPP2* in RGS14-mediated SSC proliferation, whether other downregulated genes also play a role is unclear, and we do not know whether RGS14 is directly involved in the transcriptional regulation of *PLPP2* or indirectly affects its level via other pathways. In addition, as RGS14 is a scaffolding molecule, we will study the reciprocal molecules of RGS14 in combination with protein immunoprecipitation and mass spectrometry experiments in the future to elucidate the detailed mechanism of its role.

By performing scRNA-seq analysis and cellular experiments, we discovered that RGS14 is downregulated in patients with NOA, which impacts SSC proliferation. However, conclusive evidence linking the dysregulation of RGS14 to NOA is still lacking. Despite conducting whole-exome sequencing on numerous NOA patients, no pertinent mutation sites were identified. Given that RGS14 expression commences at the zygote stage and is linked to the embryo’s first division, mutations in RGS14 could be lethal, which might explain why we failed to detect any deleterious mutations in RGS14. In future studies, we plan to expand our screening of NOA samples and consider epigenetic factors to further explore the correlation between RGS14 and the occurrence of NOA. Additionally, creating a mouse model with conditional knockout of RGS14 in the testes will be crucial for understanding the role of RGS14 in male fertility and spermatogenesis.

## Conclusion

In our study, a systematic analysis of gene expression alterations in SSCs from NOA and normal testes was conducted through scRNA-seq. It was discovered that within SSCs, RGS14 forms a complex with GNAI3, which modulates the MAPK signaling pathway and the expression of PLPP2. This, in turn, affects cell viability and self-renewal. The dysregulation of these molecules may underlie the pathogenesis of NOA. Our findings elucidate the molecular mechanisms underlying the dysfunction of SSCs in NOA and may provide novel insights for the diagnosis and therapeutic strategies for this condition.

## Data Availability

The datasets presented in this study can be found in online repositories. The names of the repository/repositories and accession number(s) can be found in the article/Supplementary Material.
