# Supplementary material for: RGS14 binds to GNAI3 and regulates the proliferation and apoptosis of human spermatogonial stem cells by affecting PLPP2 expression and MAPK signaling
Source: Front Cell Dev Biol. 2025 Apr 25;13:1593595. doi: 10.3389/fcell.2025.1593595 (PMC12061929; doi:10.3389/fcell.2025.1593595)
Supplement: Supplementary file 1 [file Supplementaryfile1.docx]

**Supplementary information**

**RGS14 Regulates the proliferation and apoptosis of human spermatogonial stem cells through PLPP2**

**Supplemental Data:**

**2Tables**

**Supplemental Tables**

**Table S1.** The primers used for qPCR and RT-PCR

| **Genes** | **Sequence** |
| --- | --- |
| *RGS14* | **F:** CGCGGAAAACGTGACTTTCTG |
|  | **R:** CTGACGGTCGATGTTCACTGG |
| *GABRR2* | **F:** GAGTGGACGAGCACGACTTC |
|  | **R:** GCAGGTACAGGGTCATAGTGA |
| *MNS1* | **F:** TGCGGCAACGTGAAGATTTG |
|  | **R:** GCAGCCTGTAGCACTAATTCC |
| *HMGN5* | **F:** CAGGTCAAGGTGATATGAGGCA |
|  | **R:** GCTTGGGCACTTGTATCTATGT |
| *NBL1* | **F:** GGAATCCGAGCTGGACCTAAA |
|  | **R:** CCTGAAGCAAATCGACCACAG |
| *MFSD3* | **F:** ACTGTGGAATGGTGTGGGTG |
|  | **R:** GAAGACCAAGGCAGTCTGACA |
| *SNAI3* | **F:** ACTGCGACAAGGAGTACACC |
|  | **R:** GAGTGCGTTTGCAGATGGG |
| *PLPP2* | **F:** TGGAACCTACTGAACGACCTC |
|  | **R:** ATCCAAACGATCCATGTGGCA |

**Table S2.** Antibodies applied in Western blots, immunofluorescence

| Antibodies | Source | Dilution | Incubation |
| --- | --- | --- | --- |
| *Western blot* |  |  |  |
| RGS14 | Zenbio cat# 123015 | 1:1000 | 12h at 4℃ |
| PLZF | SantaCruz cat#sc-28319 | 1:1000 | 12h at 4℃ |
| PCNA | Abcam cat#ab29 | 1:500 | 12h at 4℃ |
| ERK1/2 | Promab cat#30014 | 1:1000 | 12h at 4℃ |
| p-ERK1/2 | Zenbio cat#R380698 | 1:1000 | 12h at 4℃ |
| MEK | Promab cat#P22715 | 1:1000 | 12h at 4℃ |
| p-MEK | Promab cat#P20128 | 1:1000 | 12h at 4℃ |
| ACTB | Promab cat#20270 | 1:2000 | 12h at 4℃ |
| GFRA1 | R&D cat#AF560 | 1:500 | 12h at 4℃ |
| PLPP2 | Antibodies-online cat# ABIN7163353 | 1:1000 | 12h at 4℃ |
| *Immunofluorescence* |  |  |  |
| RGS14 | Zenbio cat# 123015 | 1:50 | 16h at 4℃ |
| GFRα1 | R&D cat#AF560 | 1:25 | 16h at 4℃ |
| KIT | R&D cat#AF332 | 1:25 | 16h at 4℃ |
